# Left Subclavian Vein Stenosis Secondary to Central Venous Access Placement: A Case Report

**DOI:** 10.7759/cureus.81186

**Published:** 2025-03-25

**Authors:** Abraham U González Martínez, Diomedes Durango, Cristian F Gonzalez, Luis A Ramírez Riva Palacio, Marco A González Martínez

**Affiliations:** 1 General Surgery, Instituto de Seguridad y Servicios Sociales de los Trabajadores al Servicio de los Poderes del Estado de Puebla (ISSSTEP), Puebla, MEX; 2 General Surgery, Instituto de Seguridad y Servicios Sociales de los Trabajadores del Estado, Puebla, MEX; 3 Internal Medicine, Instituto de Seguridad y Servicios Sociales de los Trabajadores del Estado, Puebla, MEX

**Keywords:** angioplasty, central venous catheters, percutaneous transluminal balloon angioplasty, stenosis, subclavian vein, thrombophilia

## Abstract

Venous stenosis secondary to central venous access is a well-recognized complication. However, in patients without kidney disease or those not undergoing hemodialysis, central venous stenosis is less frequently reported, suggesting that while the condition is documented in these specific populations, it may be underreported in the general population with other characteristics.

This study describes a 69-year-old patient with a history of thrombophilia due to MTHRF 677 gene heterozygous mutation, who developed left subclavian vein stenosis two weeks after the use of central vascular access, leading to left upper extremity edema, development of collateral venous network, and pain and dyspnea. Endovascular treatment was performed with recanalization and angioplasty of the subclavian vein, with favorable outcomes, improving symptoms in the immediate post-operative period and continuing through six-month follow-up with monthly consultation and phlebography control. The adequate response to this type of clinical situation with endovascular management and monotherapy with anticoagulant factor Xa inhibitor (apixaban) confirms this approach as another alternative within the field of vascular surgery.

## Introduction

Stenosis or obstruction of a thoracic central vessel can affect intrathoracic segments such as the subclavian vein, brachiocephalic vein, internal jugular veins, and the superior vena cava. These central veins are characterized by large diameters, few valves, and a high-flow system compared to the peripheral venous system, such as the superficial venous system of the lower limbs. Venous stenosis secondary to venous access is rare in the literature, although multiple cases of catheter access for hemodialysis have been documented, associating the catheter material and its duration with the development of stenosis [1]. Regarding thrombosis episodes in subclavian central access, 15% are symptomatic, and their etiology is multifactorial, with key factors including the insertion technique, catheter size, material, and duration of use [2].

The triggering mechanism generally involves endothelial damage, followed by vessel wall involvement, leading to microthrombi, smooth muscle proliferation, and eventually stenosis. Clinically, patients exhibit symptoms such as localized pain, edema, and the development of collateral venous network signs typically associated with venous insufficiency. Currently, medical and surgical management recommends balloon angioplasty with or without venous stent placement, accompanied by anticoagulation therapy (with no specific type), and follow-up with angiotomography and phlebography control. Here, we present a case of a patient diagnosed with left subclavian vein stenosis secondary to central venous access placement, treated endovascularly with diagnostic phlebography and subsequent balloon angioplasty.

## Case presentation

The patient is a 69-year-old woman with a history of hypertension and thrombophilia due to the methylenetetrahydrofolate reductase (MTHFR) 677 heterozygous mutation. She was on treatment for arterial hypertension with enalapril 10 mg orally every 24 hours and secondary thrombophilia with factor Xa-inhibitor anticoagulant (apixaban) 5 mg orally every 24 hours.

In 2014, she experienced thrombotic events in the humeral artery, requiring catheterization and subsequent vascular exploration due to persistent symptoms. In 2020, she required central vascular access placement, which remained in situ for three days. She then developed progressive symptoms, initially presenting with the development of a collateral venous network in the anterior thorax, predominantly on the left side. The appearance of edema in the left upper extremity was intermittent. However, the symptoms began to affect the patient's lifestyle when the edema caused difficulty in abduction movement of the left upper extremity and dyspnea in decubitus position that affected her sleep pattern until evaluation by the Angiology, Vascular, and Endovascular Surgery service at the Institute of Social Security for Workers of the State of Puebla. An angiotomography of the supra-aortic trunks showed a decrease in the caliber of the left subclavian vein. A summary of symptom presentation and progression is shown in Table 1.

**Table 1:** Timeline of symptom progression until treatment. LUE: left upper extremity

Time	0-3 days	Fourth day	Week 4	12 months	Year 2	Year 3
Leading symptoms	Placement of central access	Central access removal	Development of collateral venous network in left anterior thorax	Edema and difficulty in LUE movements	Dyspnea in decubitus position	Evaluation an intervention

**Figure 1:**
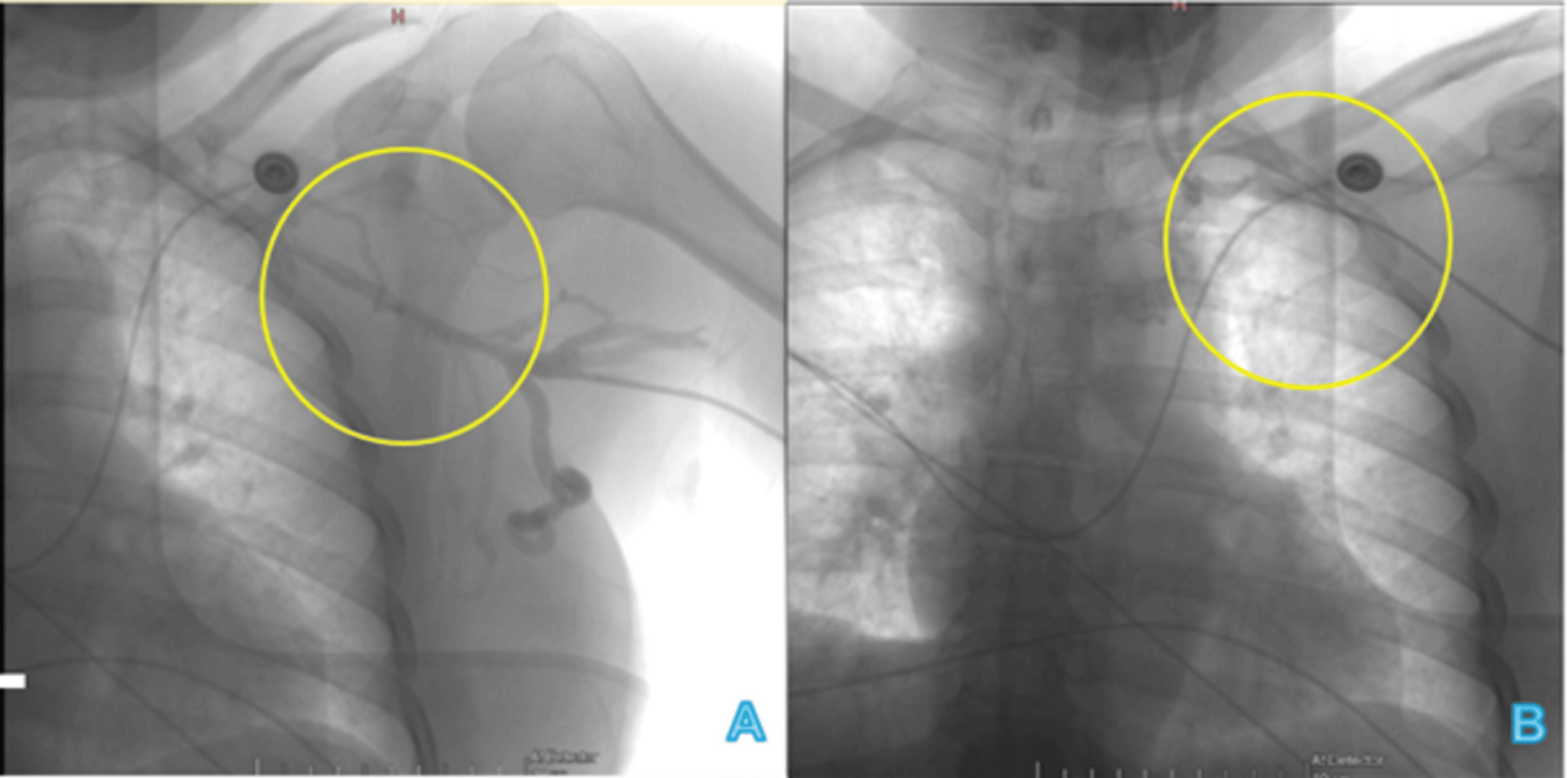
Initial left subclavian vein phlebography (A) and first approach through left brachial vein (B). The yellow circles indicate the subclavian region with stenosis (A) and the approach of the guide catheter (B).

The patient was taken for endovascular procedure, which included phlebography, recanalization, and balloon angioplasty of the left subclavian vein through the left basilic vein. The contrast medium revealed subclavian occlusion, which was recanalized by advancing a guidewire to the superior vena cava, followed by balloon angioplasty with a 3x60 mm balloon (Figures 1A, 1B).

**Figure 2:**
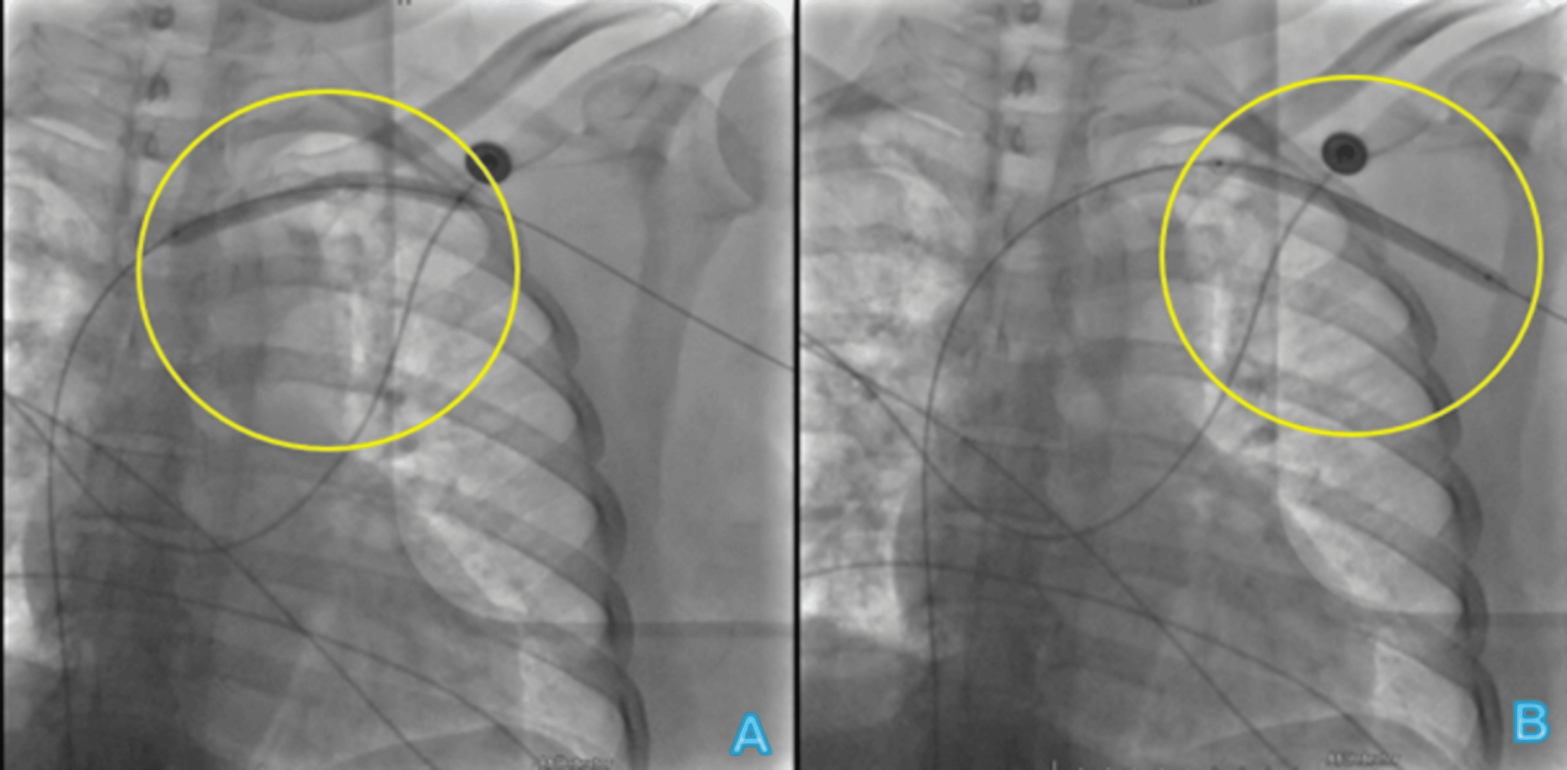
Balloon dilations via left femoral access (A) and left brachial vein (B). The yellow circles indicate the balloon angioplasty areas.

A second access was performed through the left common femoral vein to advance a hydrophilic guidewire to the left brachiocephalic vein, demonstrating the occlusion and recanalizing it using the "through-and-through" technique. A guide was advanced from the brachial access and retracted into the femoral introducer, through which 4x80 mm, 6x80 mm, and 8x10 mm balloons were used for multiple dilatations from the axillary vein to the subclavian vein, with significant improvement in contrast flow towards the superior vena cava (Figures 2A, 2B).

During this time, the patient was followed up with a phlebography during the first month after angioplasty and another phlebography at six months. The patient showed improvement in symptoms from the immediate post-operative period and continued to improve with phlebography control at six months.

## Discussion

This is a case of left subclavian vein stenosis secondary to central venous access placement via anatomical references, where the purpose was not hemodialysis therapy, which is the common cause associated with this type of stenosis. The 3SITES study reports that subclavian access has a low risk of infection and thrombosis, although mechanical complications, mainly pneumothorax, have been reported [3]. In central subclavian accesses, thrombosis episodes are symptomatic in 15%, and their etiology is multifactorial, with insertion method, catheter size, composition, and duration being the main factors to consider [4].

Physical examination is crucial, as the development of a collateral venous network in the anterior thorax and upper extremity edema is highly suggestive of venous hypertension secondary to stenosis or occlusion, specifically of the subclavian vein.

Another important point is the patient's history of thrombophilia with limited documentation in the Mexican population. This involves the heterozygous MTHFR C677T mutation, which leads to the substitution of alanine for valine at amino acid 677. Elevated homocysteine levels have been shown to increase thrombin activity, which leads to heightened activity of factors XII and V, inhibiting thrombomodulin expression. MTHFR 677-related mechanisms increase the risk of venous and arterial thrombosis, including endothelial toxicity, platelet activation, oxidation of low-density lipoproteins, and increases in von Willebrand factor and factor VII [5].

Regarding surgical management, endovascular treatment with simple balloon angioplasty was chosen. It is well-known that balloon angioplasty modifies focal stenoses in the subclavian vein, as documented in the images obtained during the procedure. Balloon angioplasty was performed for a duration of 2-3 minutes between each balloon used, leading to improvement in post-angioplasty projections.

Recurrence rates for stenosis in such cases range from 10% to 43% within a year. However, given the improvement observed in the surgical event, confirmed by phlebographic control, management without stent placement was chosen [6].

According to the KDOQI guidelines, balloon angioplasty as primary management in clinically and angiographically significant lesions is reasonable [7]. This is compounded by the patient's thrombophilia, where stenting is associated with a high probability of early occlusion [8].

## Conclusions

Subclavian vein stenosis secondary to central venous access is an underreported complication. The primary complications associated with such access include pneumothorax, infection, and thrombosis. This study presents an organized approach, beginning with diagnostic phlebography followed by endovascular therapy, without venous stent placement.

Documenting such cases is important, as central venous accesses are commonly used in clinical practice, such as for chemotherapy administration. When choosing an access type, patient characteristics, sepsis status, history of venous thrombosis, cancer, thrombophilia, prolonged rest, and high likelihood of prolonged hospitalization should be considered. The systematic approach demonstrated in this study, with short- and medium-term follow-up, shows that balloon dilation alone in endovascular therapy is a suitable treatment option for venous stenosis unrelated to hemodialysis therapy, but associated with the thrombophilia factor due to the methylenetetrahydrofolate reductase (MTHFR) 677 heterozygous mutation. We believe this represents an area of opportunity for endovascular treatments.
